# Juvenile dermatomyositis with central nervous system involvement: two case reports from a retrospective single-center cohort, with literature review

**DOI:** 10.3389/fped.2024.1409950

**Published:** 2024-05-30

**Authors:** Ling Yang, Wanzhen Guan, Haimei Liu, Yifan Li, Yinv Gong, Qianying Lv, Qiaoqian Zeng, Qijiao Wei, Xiaomei Zhang, Weiming Chen, Chao Chen, Li Sun

**Affiliations:** ^1^ Department of Rheumatology, Children’s Hospital of Fudan University, Shanghai, China; ^2^ Department of Rheumatology, Children’s Hospital of Fudan University at Xiamen (Xiamen Children’s Hospital), Fujian, China; ^3^ National Children’s Medical Center, Shanghai, China; ^4^ Pediatric Intensive Care Unit, Children’s Hospital of Fudan University, Shanghai, China; ^5^ Department of Otolaryngology, Children’s Hospital of Fudan University, Shanghai, China

**Keywords:** juvenile dermatomyositis, anti-NXP2 antibody, cutaneous vasculitis, seizure, macrophage activation syndrome, plasma exchange, outcomes

## Abstract

**Background:**

Juvenile dermatomyositis (JDM) is a systemic autoimmune disease primarily involving the muscles and skin; it can also affect the central nervous system (CNS). The relevant literature provides limited information regarding the characteristics of JDM with CNS involvement.

**Method:**

We reviewed patients with JDM who were hospitalized at our center between January 2016 and August 2023, with a focus on those with CNS involvement. The aim was to provide detailed case reports on these patients, and to summarize the relevant literature about the characteristics of similar cases.

**Results:**

Among 193 hospitalized patients with JDM, two (1.03%) had CNS involvement. Two patients, a 5.5-year-old girl and an 11-year-old boy, were admitted with severe proximal muscle weakness and seizures, and presented with active cutaneous vasculitis. Both were ultimately diagnosed with JDM, with CNS involvement. Both patients had confirmed presence of anti-NXP2 antibody through myositis-specific antibody analysis. Additionally, they all exhibited hyperferritinemia and thrombocytopenia. Salvage therapies like intravenous methylprednisolone (IVMP) pulse therapy and/or plasma exchange were administered successfully. At final follow-up, both patients had achieved complete clinical response and full neurological recovery. Our literature review identified nine similar case studies. CNS involvement usually occurred within the first 10 months of the disease course, and most of these patients had fatal outcomes, with a mortality rate of 66.6% (6/9). Including the two patients described herein, the median age for disease onset is 10.5 years (range 4–17 years), and the male: female ratio is 6:5. Seizures are the most common neurological symptom, accompanied by active cutaneous vasculitis. The brain biopsies showed two distinct pathological presentations: one was central nervous system vasculitis, and the other was cerebral macrophage activation syndrome.

**Conclusions:**

CNS involvement is a rare but life-threatening JDM complication. Herein, our cases and the literature indicate that it typically occurs within the first 10 months of the disease course and manifests as seizures, often accompanied by active cutaneous vasculitis, with fatal outcomes. Timely implementation of salvage therapies, like IVMP pulse therapy and plasma exchange, may significantly impact patient outcomes.

## 1 Introduction

Juvenile dermatomyositis (JDM) is a rare systemic autoimmune disease characterized by systemic capillary vasculopathy, primarily affecting the skin and skeletal muscles, with potential involvement of other organs, including the central nervous system (CNS) (1, 2). CNS involvement in JDM is a life-threatening complication. Recognizing and understanding neurological complications are crucial for accurate diagnosis, effective management, and improved outcomes in patients with JDM. However, in contrast to other autoimmune diseases in childhood (e.g., systemic lupus erythematosus), reports of CNS involvement in JDM are extremely rare (3, 4). This under-recognition of CNS involvement in JDM challenges early diagnosis and appropriate treatments.

The precise pathogenesis underlying CNS involvement in JDM is not yet fully understood. However, it is believed to result from a combination of immune-mediated processes, vascular abnormalities, and direct invasion of inflammatory cells into the CNS. Recent advancements have shown that specific serum antibodies for JDM, known as myositis-specific antibodies (MSA), are closely associated with organ involvement or clinical presentations in various JDM subtypes (5, 6). However, to date there have been no reports or studies specifically associating MSAs with CNS involvement.

Herein, we conducted a retrospective analysis of patients with JDM who were hospitalized at our center, focusing specifically on those with CNS involvement to provide detailed case reports. We also analyzed MSA results from all patients to determine whether any were associated with CNS involvement. Finally, we summarize the literature on clinical features of patients with JDM with CNS involvement.

## 2 Materials and methods

### 2.1 Study sample

The study was approved by Children’s Hospital of Fudan University's Ethics Committee [2023] (No. 277). All patients with JDM who were admitted to the Department of Rheumatology, Children's Hospital of Fudan University, between August 2016 and August 2023, were included in this retrospective study. The inclusion criteria were: age <18 years; and JDM diagnosis based on either the 1975 Bohan and Peter criteria or the 2017 European League Against Rheumatism/American College of Rheumatology (EULAR/ACR) classification (7). Initial MSA analyses for all patients with JDM were collected and case reports were developed for those with CNS involvement.

### 2.2 Search strategy and data extraction

A comprehensive electronic search strategy of databases, including PubMed and China National Knowledge Infrastructure, was performed for data published during the past 30 years (between 1993 and August 2023) with title, abstract, or keyword terms: (“dermatomyositis” OR “juvenile dermatomyositis”) AND (“central nervous system” OR “vasculitis, central nervous system” OR “encephalopathy” OR “seizures”). Samples of patients under age 18 years were included, and those articles were included in the full-text review. Patients with overlapping syndromes and those considered to have no relation between the neurological symptoms and dermatomyositis were excluded from the analysis.

The following data were extracted from the included studies: (1) Descriptive information, including country, publication year, gender, and age; (2) Clinical characteristics, including fever, cutaneous vasculitis status, and neurological symptoms; (3) Laboratory indicators, including blood routine examination, serum ferritin, and initial creatine kinase (CK) levels, and MSA analysis results; (4) Neurological examination, including cerebrospinal fluid examination, brain imaging, and brain biopsy; (5) Post-event treatments and outcomes.

### 2.3 Statistical analysis

Sample characteristics are summarized by descriptive statistics. Frequencies are expressed as “*n*” or “*N*”, and ratios as percentages.

## 3 Results

### 3.1 Center patent sample

In our center, 193 patients met the inclusion criteria. Among 193 patients with JDM, two were identified as having CNS involvement, indicating an incidence rate of 1.03% (*N* = 2, 2/193). Among the overall sample, 175 patients had MSA analysis, with the following results (Table 1): 116 patients tested positive (*n* = 116, 66.3%). The main positive MSA were NXP2 (*n* = 44, 25.1%), MDA5 (*n* = 42, 24.0%), TIF-1γ (*n* = 26, 14.9%), SAE (*n* = 3, 1.7%), and Mi-2 (*n* = 1, 0.6%). Both patients with CNS involvement tested positive for anti-NXP2 antibody. The incidence of CNS involvement in patients who were anti-NXP2 antibody-positive was 4.54% (*N* = 2, 2/44).

**Table 1:** Myositis-specific antibody analysis and central nervous system involvement in case study patients.

	Frequency *n* (%)				CNS involvement frequency *N* (*N*/*n*%)
Total	193 (100)				2 (1.03)
MSA analysis	175 (90.7)	Negative		59 (33.7)	0
		Positive	NXP2	44 (25.1)	2 (4.54)
			MDA5	42 (24.0)	0
			TIF-1γ	26 (14.9)	0
			SAE	3 (1.7)	0
			Mi-2	1 (0.6)	0
Other (not available/not tested)	18 (9.3)				0

### 3.2 Patient descriptions

#### 3.2.1 Case 1

**Figure 1:**
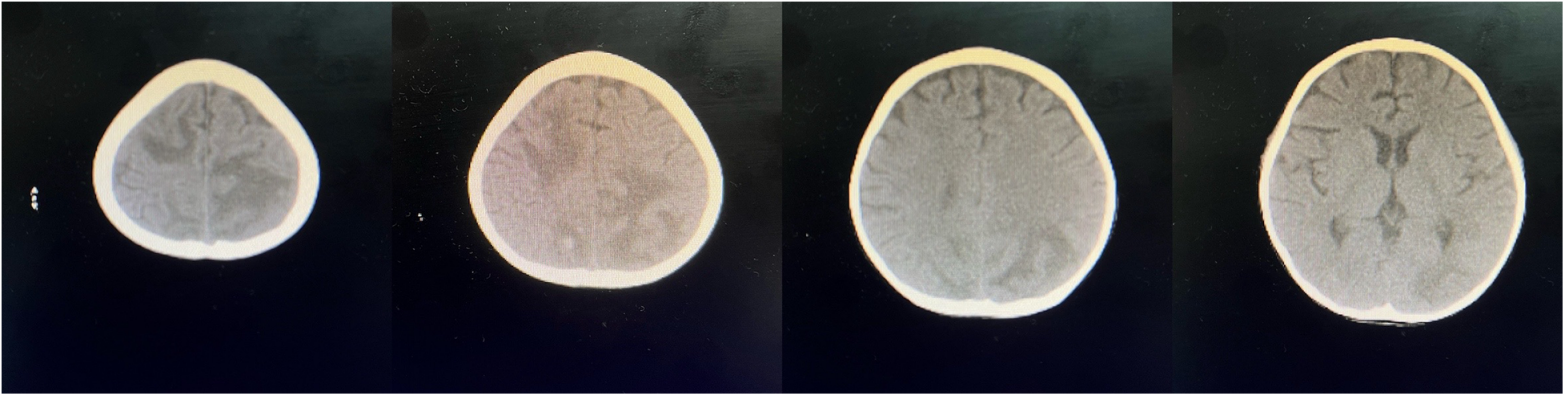
Case 1 cerebral CT demonstrated multiple bilateral frontal and parietal lobe lesions with low density, without evidence of intracranial bleeding.

**Figure 2:**
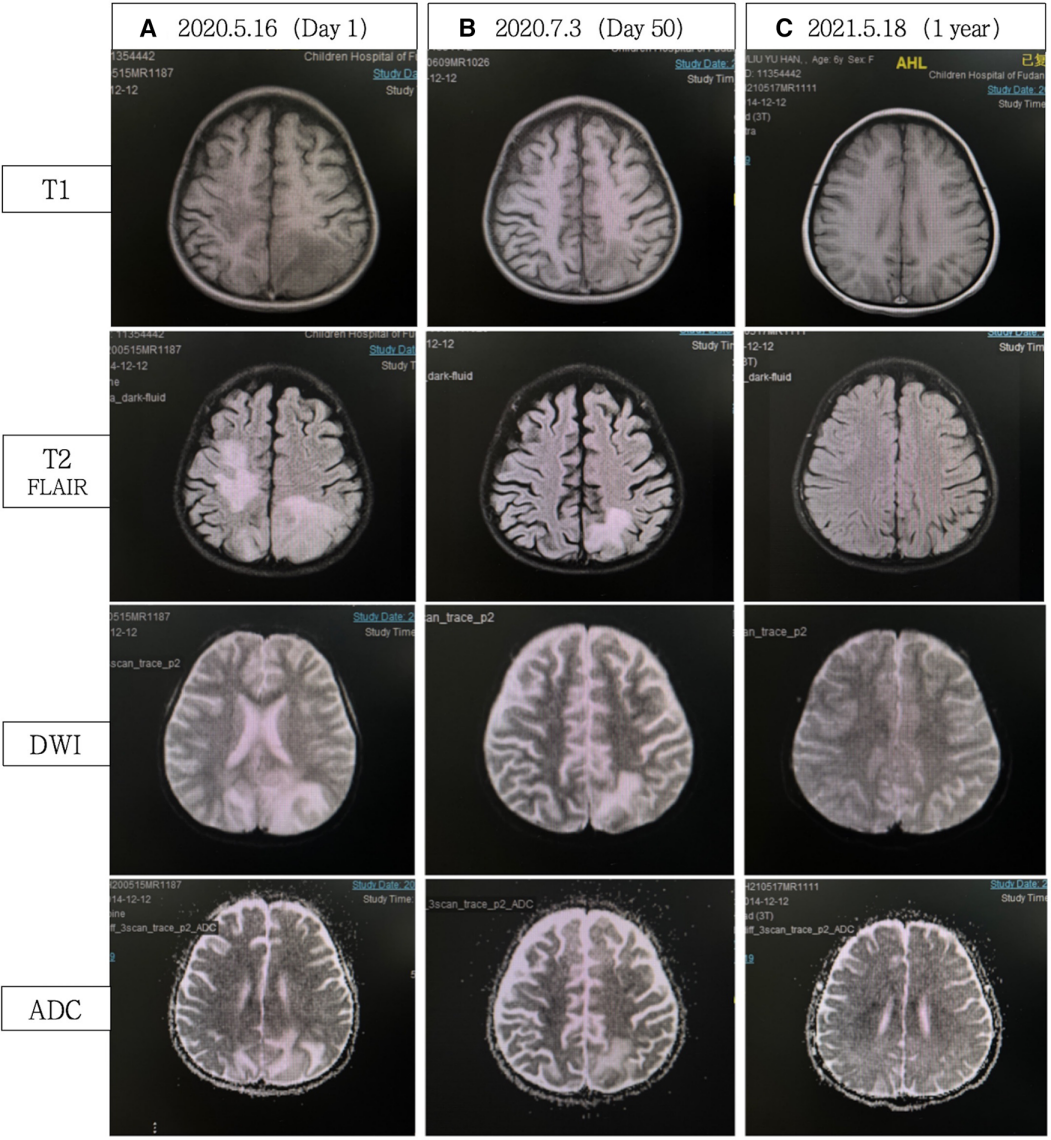
(**A**) Cerebral MRI demonstrated extensive bilateral T2 FLAIR/DWI hyperintense and T1 hypointense lesions, without ADC value reduction. (**B**) Significant reduction in abnormal signals compared with A. (**C**) Complete resolution of abnormal signals. FLAIR, fluid attenuated inversion recovery; DWI, diffusion weighted imaging; ADC, apparent diffusion coefficient.

A 5.5-year-old girl presented with a one-month history of proximal muscle weakness and dysphagia. Cerebral computed tomography (CT) scan showed multiple bilateral frontal and parietal lobe lesions with low density, without evidence of intracranial bleeding (Figure 1). The child was quickly transferred to the pediatric intensive care unit (PICU). Her previous medical history was unremarkable. Upon admission, the child showed symptoms of generalized seizures without fever, followed by a depressed conscious state. Her blood pressure was 101/73 mmHg. Mild red rashes were observed on her face, trunk, and limbs. There were no enlarged lymph nodes or hepatosplenomegaly. Limb muscle strength was graded as 0–1/5. Hematological examination showed elevated muscle enzyme markers, including aspartate aminotransferase (AST, 238 U/L, reference range: 14–44 U/L), alanine aminotransferase (ALT, 159 U/L, reference range: 7–30 U/L), lactate dehydrogenase (LDH, 950 U/L, reference range: 110–290 U/L), and CK (180 U/L, reference range: 0–164 U/L). White blood cell count was 27.3 × 10^9^ /L with 50.1% neutrophils. Hemoglobin was 101 g/L and platelet count was 31 × 10^9 ^/L. C-reactive protein and erythrocyte sedimentation rates were within the normal range. Serum ferritin (1,014 ng/ml, reference range: 9.94–71.7 ng/ml) was significantly elevated, indicating hyperferritinemia. Triglyceride was 8.95 mmol/L (reference range: 0–1.7 mmol/L). She had no coagulation disorders. Serum electrolytes and renal function were within the normal ranges. Blood and urine tandem mass spectrometry were negative. Antinuclear antibody was 1:100 positive, anti-extractable nuclear antigen antibody was negative, and complement was normal. Multiple blood cultures were negative. A lumbar puncture was performed within 24 h of admission. Spinal fluid protein, glucose, and cell counts were within normal limits, and culture was negative. Bone marrow aspiration revealed hemophagocytosis. However, the diagnosis of hemophagocytic lymphohistiocytosis (HLH) based on the HLH-2004 classification criteria was insufficient. Echocardiography and electrocardiogram were normal. Chest x-ray revealed slight patchy opacities. Cerebral magnetic resonance imaging (MRI) revealed extensive T2 hyperintense and T1 hypointense bilateral lesions (Figure 2A). Cerebral magnetic resonance angiography and venography (MRA and MRV, respectively), and full-spine MRI, did not show any significant abnormalities. Electroencephalogram (EEG) showed diffuse slow waves with a frequency of 2–4 Hz.

She was treated post-admission with broad-spectrum antibiotics to cover a possible infection. Temporary sedation and intracranial pressure management were implemented to alleviate neurological symptoms. However, on hospitalization day 3 she developed respiratory distress and mechanical ventilation was initiated. Autoimmune encephalitis and CNS inflammatory demyelinating disease could not be ruled out as the cause of her clinical state. She received intravenous immunoglobulin (IVIG) therapy. However, the results of the cerebrospinal fluid analysis for autoimmune encephalitis and CNS inflammatory demyelinating disease-associated antibodies were negative. Simultaneously, asymmetrical upper limb edema with reticulated purpura was noted, while venous doppler ultrasound did not indicate any signs of thrombotic events. Excluding the possibility of CNS infections, hypoperfusion/hypertensive encephalopathy, electrolyte and metabolic abnormalities, hypercoagulability, CNS inflammatory demyelinating disease, and autoimmune encephalopathy, we suspected JDM based on the presence of elevated CK levels, severe muscle weakness, and rash. Her Childhood Myositis Assessment Scale (CMAS) score was 4 points. Consequently, further evaluation was conducted, including MSA analysis, thigh MRI, electromyography, and muscle biopsy.

**Figure 3:**
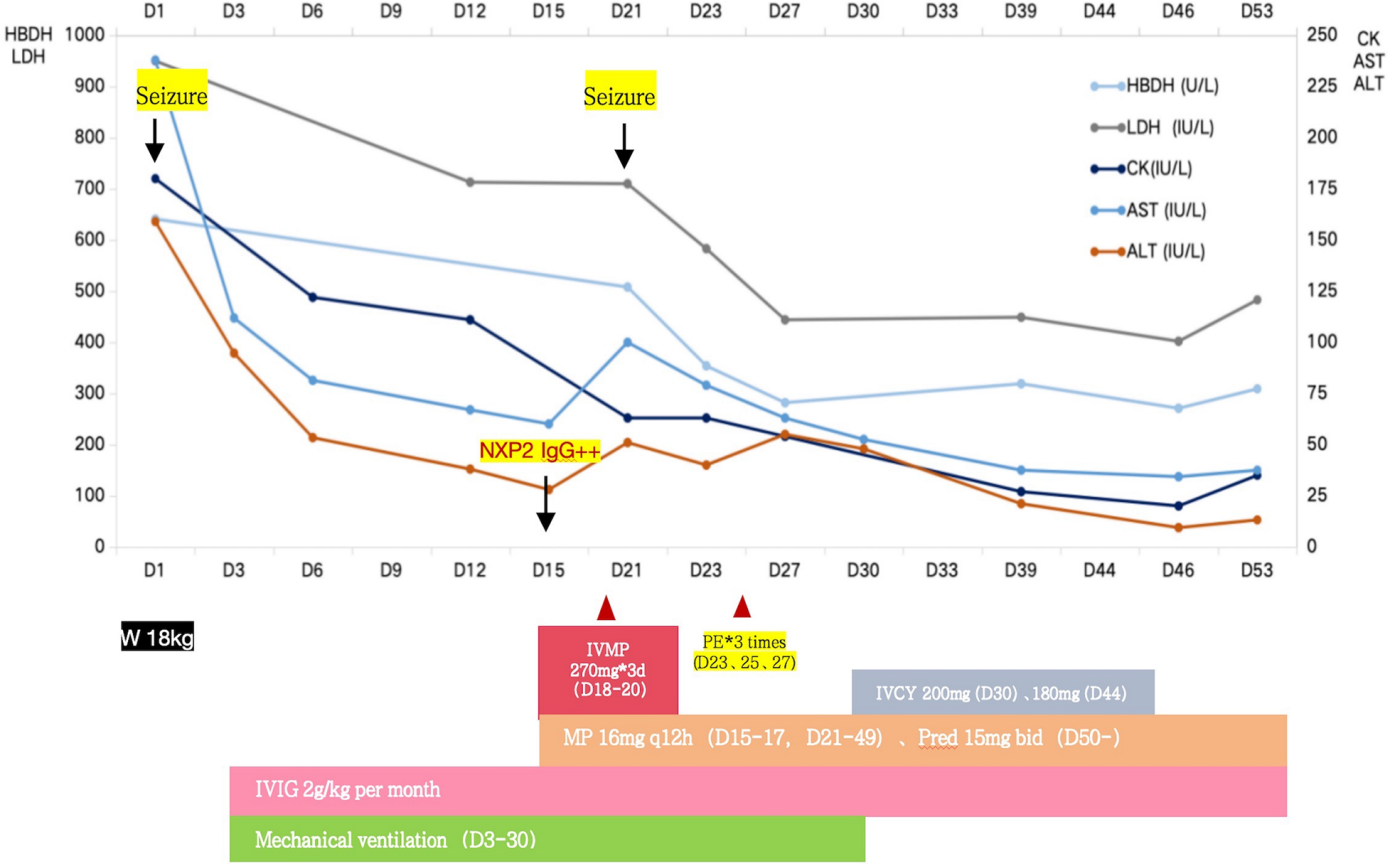
Case 1 clinical course.

Thigh MRI revealed diffuse abnormal signals in the soft tissues and muscles of both thighs and buttocks. Electromyography indicated myogenic damage. Muscle biopsy showed vacuolar muscle fibers, muscle fiber atrophy, and scattered or clustered mononuclear inflammatory cells around the endomysium, perimysium, and some small blood vessels. Immunohistochemical staining for major histocompatibility complex class I (MHC I) showed widespread positive reactivity on the muscle membrane, consistent with myositis changes. Serum MSA analysis confirmed the co-presence of anti-NXP2 antibody and anti-Ro-52 antibody. On hospitalization day 15, the patient was diagnosed with definite idiopathic inflammatory myopathy, per the 2017 EULAR/ACR criteria, with a total aggregate score of 12.3 > 8.7. Despite the absence of typical dermatomyositis skin rash, she was classified as having JDM based on her rare skin manifestations, such as asymmetric upper limb edema and reticular purpura, along with the MSA analysis results. CNS involvement related to JDM was considered the cause of seizures. As shown in Figure 3, the patient was started on intravenous methylprednisolone (IVMP, 1.6 mg/kg/day). Additionally, IVMP pulse therapy (15 mg/kg/day) was administered for three days (hospitalization days 18–20). Monthly maintenance IVIG was continued at a dose of 2 g/kg. Because of a lack of significant muscle strength improvement and seizure recurrence on hospitalization day 21, plasma exchange (PE) was performed on hospitalization days 23, 25, and 27 (i.e., three total sessions). Intravenous cyclophosphamide (IVCY) therapy was subsequently added at doses of 200 mg on hospitalization day 30 and 180 mg on hospitalization day 44. After that, the patient no longer experienced seizures, her respiratory status stabilized, and her muscle strength improved. She was successfully weaned off ventilation on hospitalization day 30. Her anti-NXP2 antibody level was 110.29 RU/ml (reference range <40 RU/ml) on hospitalization day 35. Cerebral MRI on hospitalization day 50 revealed significantly reduced abnormal signals (Figure 2B) and repeated EEG also showed brainwave activity improvement. The patient was successfully discharged on hospitalization day 53.

Steroid therapy was continued post-discharge. Due to pulmonary infection, cyclophosphamide was discontinued, with a total accumulated dose of 580 mg. Sequential oral mycophenolate mofetil and monthly maintenance IVIG (2 g/kg) were administered. After eight months of this therapy course, CMAS fluctuated from 26 to 31 and facial flushing was observed; at that time, tofacitinib was added. The most recent follow-up was January 2024 (3.5 years into the disease course), when steroids had been discontinued for one year and mycophenolate mofetil for two months, and with continued tofacitinib monotherapy. At that time, the child maintained clinical remission from JDM without neurological symptoms. Her NXP2 antibody titer IgG had decreased to 20 RU/ml and her cerebral MRI remained stable throughout follow-up, similar to the results shown in Figure 2C.

#### 3.2.2 Case 2

An 11-year-old boy presented with a one-month history of proximal muscle weakness and a typical heliotrope rash, and was admitted to our hospital in July, 2022. His CMAS score was 12. Serum CK was elevated (4,718 U/L, reference range: 0–164 U/L). Electromyography indicated myogenic damage. Muscle biopsy showed typical perifascicular atrophy, inflammatory cells primarily distributed around the muscle fascicles, endomysium, and small blood vessels, and extensive positive staining of MHC I on the muscle membrane. MSA analysis confirmed the presence of anti-NXP2 antibody and anti-Ro-52 antibody. Anti-NXP2 antibody IgG level was 115.42 RU/ml, with ANA 1:320 (+). He was diagnosed with definite JDM according to 2017 EULAR/ACR criteria with a total aggregate score 14.9 > 8.7. Considering the critical condition of the patient, he was initiated on IVIG at a dose of 2 g/kg and IVMP at 500 mg/day for three days, followed by oral prednisone at 2 mg/kg/day. A dose of IVCY at 500 mg was also administered.

**Figure 4:**
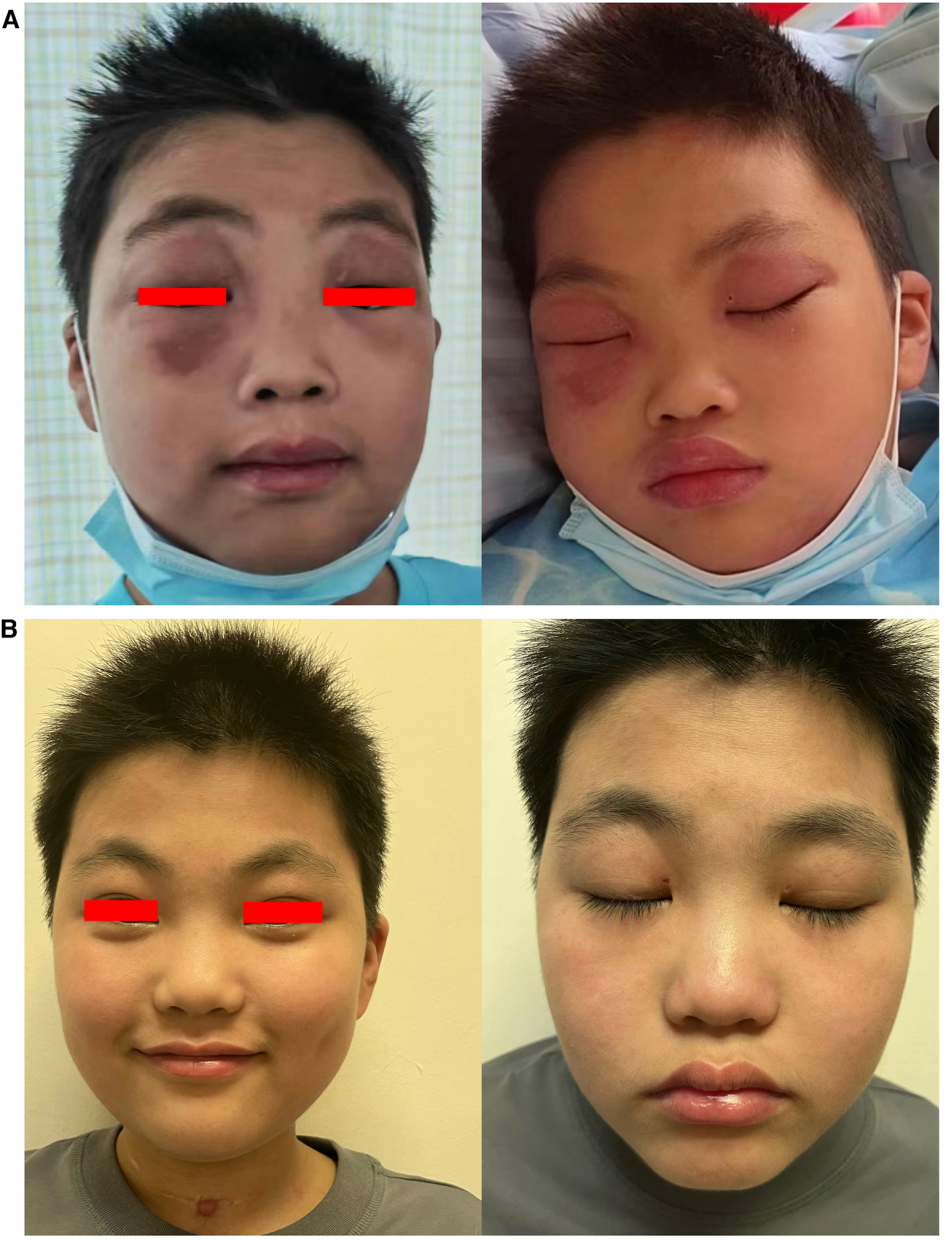
(**A**) Periorbital edema before treatment and (**B**) after one year of treatment.

**Figure 5:**
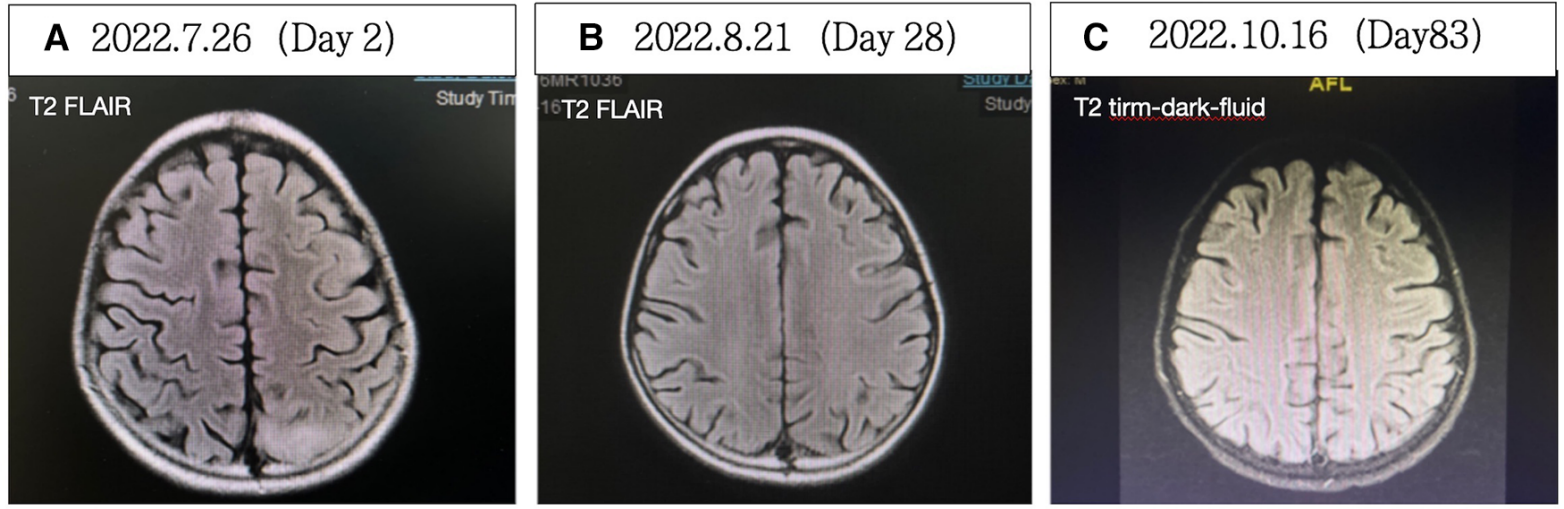
(**A**) Contrast-enhanced cerebral MRI showing abnormal signals in the left parieto-occipital lobe and thalamus on hospitalization day 2. (**B**) Cerebral MRI revealed less pronounced abnormal signals in the left parieto-occipital lobe and thalamus on hospitalization day 28. (**C**) No significant abnormal signals in the brain parenchyma were observed on hospitalization day 83.

After treatment, muscle weakness persisted, and the patient gradually developed dysphagia. Two weeks after treatment, he suddenly experienced tonic-clonic seizures and remained in a coma between seizures, indicating status epilepticus. Sedative medications and assisted ventilation via endotracheal intubation were administered, and he was promptly admitted to the PICU. Physical examination revealed significant skin and periorbital edema (Figure 4A). Blood pressure was 126/81 mmHg. Cardiac and pulmonary physical examination showed no abnormalities. There were no enlarged lymph nodes or hepatosplenomegaly. Positive Babinski sign was observed bilaterally, while other pathological signs were negative. Laboratory examinations revealed elevated levels of AST (269 U/L), ALT (76 U/L), LDH (1,200 U/L), and CK (4,784 U/L). White blood cell count was 17.15 × 10^9 ^/L, with 91.4% neutrophils. Hemoglobin level was 137 g/L, and platelet count decreased to 39 × 10^9^ /L. C-reactive protein level was within normal range. Serum ferritin was significantly elevated (>2,000 ng/ml), indicating hyperferritinemia. Triglyceride was 2.56 mmol/L. Serum electrolytes were in the normal range. There were no coagulation disorders. NXP2 antibody titer IgG was higher than initial admission: 139.76 RU/ml. Echocardiography showed normal cardiac function. Chest CT indicated bilateral lung infiltrates and partial consolidation. Cerebral CT showed blurred gray-white matter differentiation, and contrast-enhanced cerebral MRI (Figure 5A) revealed abnormal signals in the left parieto-occipital lobe and thalamus. Cerebral MRA and MRV were negative. EEG showed diffuse background slow waves (2–5 Hz low-to-moderate amplitude slow waves). Spinal fluid studies revealed normal cell counts and glucose level, with a slight increase in protein concentration (64 mg/dl, reference range: 15–45 mg/dl), and no evidence of bacteria or viruses.

**Figure 6:**
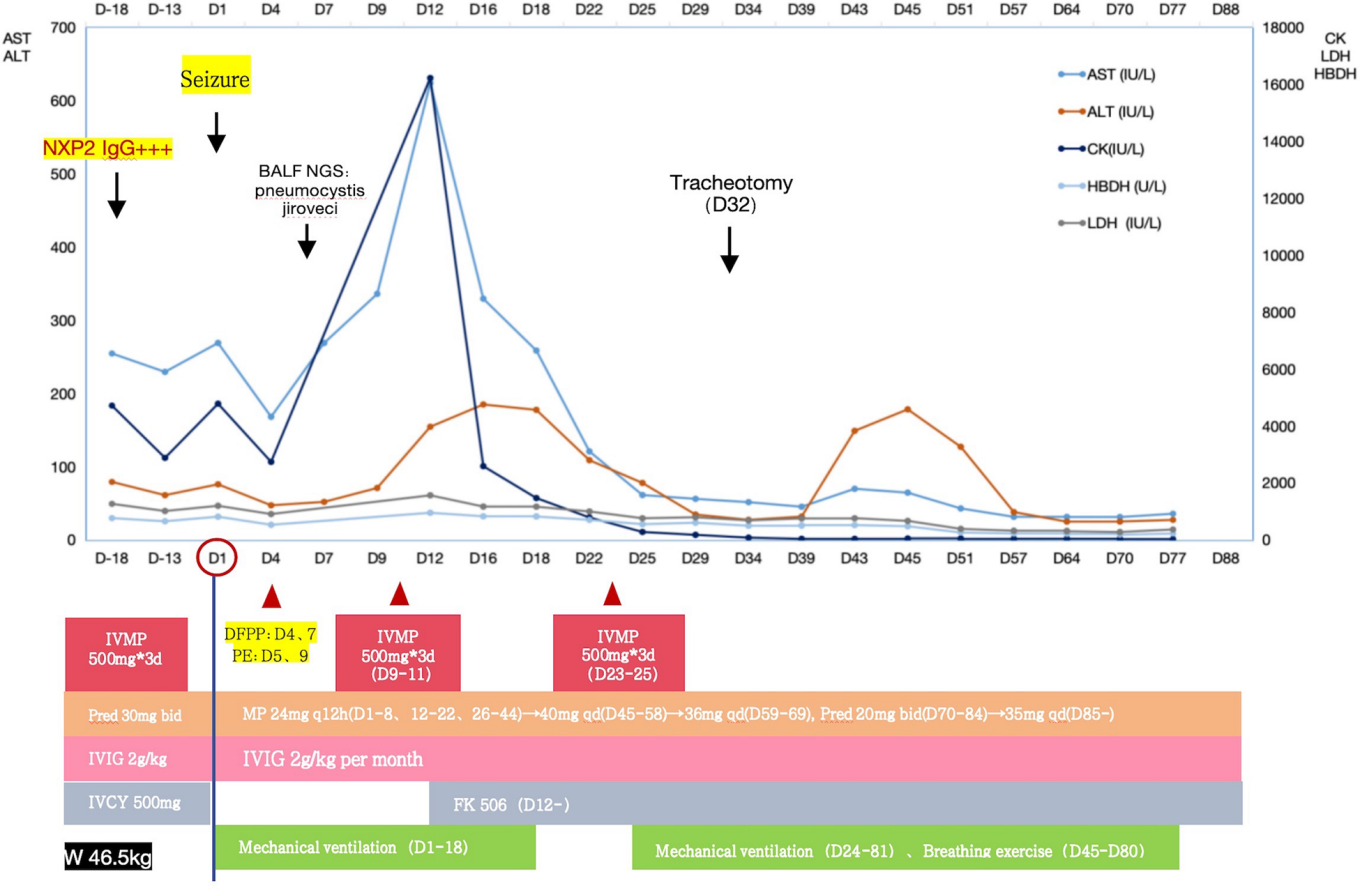
Case 2 clinical course.

After a thorough examination and analysis, considering the significant skin and periorbital edema, muscle weakness, significantly elevated muscle enzymes, and an increasing trend in NXP2 antibody titer IgG, we concluded that the patient was in the active stage of JDM, and seizures were attributed to CNS involvement. Considering the patient's worsening condition despite conventional treatment, we discussed his case and decided to initiate therapeutic PEs. As shown in Figure 6, four PEs were performed, including two sessions of double filtration plasmapheresis. IVMP (1.6 mg/kg/day) and maintenance IVIG (2 g/kg/month) was continued. In addition, pneumocystis jirovecii was detected in the metagenome next-generation sequencing analysis of bronchoalveolar lavage fluid on PICU hospitalization day 6, so antifungal treatments were administered. Despite intensive PE treatment, his condition deteriorated further, and CK levels increased to 16,219 U/L. Two additional courses of IVMP therapy (500 mg/day for three days), over a two-week interval, were administered. Considering that the patient presented with hyperferritinemia and thrombocytopenia, macrophage activation syndrome (MAS) could not be ruled out, despite the absence of fever. Therefore, oral administration of tacrolimus (i.e., FK506) was initiated. Subsequently, a bone marrow examination revealed the presence of macrophages. In addition, tracheostomy was performed because prolonged intubation was needed.

After the treatments, the patient's seizures ceased, muscle strength improved, swallowing and respiratory functions recovered, and muscle enzyme levels decreased to 41 U/L. The patient was successfully weaned off the ventilator. On hospitalization day 58, EEG showed a baseline electrical activity of 9–10 Hz α waves with some θ and δ waves. Cerebral MRI on hospitalization day 28 (Figure 5B) indicated less pronounced abnormalities, and cerebral MRI on hospitalization day 83 (Figure 5C) revealed no significant abnormalities. These findings indicated an improved patient condition and he was successfully discharged on hospitalization day 91.

After discharge, oral steroids combined with tacrolimus were continued, along with monthly maintenance IVIG at a dose of 2 g/kg. In March 2024 (20 months into the disease course), the steroid dosage had been decreased to 2.5 mg/day and assessments showed a CMAS score of 52, complete resolution of the rash (Figure 4B), no neurological symptoms, and normal muscle enzyme levels. There was also a decrease in NXP2 antibody titer IgG to 15.85 RU/ml.

### 3.3 Literature review

The resulting literature was almost exclusively case reports. A total of 13 articles (8–20) described 16 patients (adult dermatomyositis, *n* = 6; JDM, *n* = 10) with CNS involvement. From the 10 pediatric cases, one child with overlapping syndromes was excluded (14). Finally, a total of 9 pediatric cases from six articles (8–13) were identified. Combining these patients with the two from our center described herein, we conducted a descriptive analysis of the clinical characteristics of these 11 patients (Tables 2, 3).

**Table 2:** Clinical and laboratory characteristics of patients with JDM with CNS involvement.

ID	Country (year)	Age, gender	Initial CK (U/L)	MSA	Active cutaneous vasculitis	Seizure	Fever	Ferritin (ng/ml)	Blood cells
1	Canada (1994) [8]	6, F	5,440	NA	B	Yes	No	NA	NA
2	Italian (2001) [9]	4, F	<20	NA	A, C, D	Yes	NA	NA	NA
3	Afro-Caribbean (2001) [9]	10, F	NA	NA	NA	Yes	Yes	NA	NA
4	Netherlands (2003) [10]	11, M	4,260	NA	A	Yes	No	NA	NA
5	Netherlands (2003) [10]	11, M	3,923	NA	NA	Yes	No	NA	NA
6	Netherlands (2003) [10]	4, M	1,532	NA	B, C	Yes	No	NA	NA
7	Japan (2013) [11]	17, F	1,094	NA	NA	Yes	Yes	4,172	Pancytopenia
8	Norway (2014) [12]	12, M	14,000	NA	A	Yes	NA	3,889	NA
9	Indian (2022) [13]	11, M	NA	(−)	A	Yes	Yes	4,059	Pancytopenia
10	China (2022) [Case 1]	5.5, F	186	NXP2 +++	A, D	Yes	No	1,014	Thrombocytopenia
11	China (2023) [Case 2]	11, M	4,718	NXP2 +++	A	Yes	No	>2,000	Thrombocytopenia

NA, not available; CK, creatine kinase; MSA, myositis specific antibody; Active cutaneous vasculitis of (A) face/lips/eye orbit/limb edema, (B) dilated nail bed capillaries, (C) new ulcers on fingers/toes, and/or (D) reticulated purpura.

**Table 3:** Clinical and laboratory characteristics of patients with JDM with CNS involvement.

ID	Time from JDM onset to event	Cerebrospinal fluid	Brain biopsy	Brain imaging (positive test)	Post-event treatment	Outcome
1	3 days	NA	CNSV	CT (CT)	PSL	Death
2	10 days	(−)	NA	CT, MRI (MRI)	PSL, IVCY, TPE 2 times	Death
3	5 months	(−)	NA	MRI, MRA (None)	IVIG, PSL, IVCY	Survival
4	2 weeks	(−)	CNSV	CTA, ECT (CTA, ECT)	IVCY	Death
5	4 weeks	Protein 60 mg/dl	CI	CT, MRI (CT, MRI)	IVIG, IVCY	Death
6	9 weeks	(−)	NA	NA	−CyA, IVIG	Death
7	27 days	(−)	macrophage infiltration, ICH	MRI (MRI)	MPSL pulse, PSL, CyA, IVCY	Death
8	1 months	(−)	NA	MRI (MRI)	−CyA, VP16, anakinra	Survival
9	1 week	NA	NA	MRI, MRA (MRI)	−MTX, MPSL pulse, IVIG, oral CyA, IVCY, RTX	Survival
10	Onset of CNS involvement	(−)	NA	CT, MRI, MRA, MRV (CT, MRI)	MPSL pulse, PSL, IVIG, TPE 3 times, IVCY	Survival
11	2 weeks	Protein 64 mg/dl*	NA	CT, MRI, MRA, MRV (CT, MRI)	MPSL pulse, PSL, IVIG, TPE 4 times, FK506	Survival

CNSV, central nervous system vasculitis; CI, cerebral infarction; “*”, reference range: 15–45 mg/dl; “−“, discontinued; MPSL, methylprednisolone; PSL, prednisolone; MTX, methotrexate; CyA, cyclosporin A; IVIG, intravenous immunoglobulin; IVCY, intravenous cyclophosphamide; TPE, therapeutic plasma exchange; FK506, tacrolimus.

The median age of disease onset was 10.5 years (range 4–17 years). The male-to-female ratio was 6:5. Seizures were the most common clinical manifestation of CNS involvement (100%, 11/11). Seizures occurred in 10 patients within three days to 10 months after diagnosis; in one patient, symptom onset occurred before diagnosis. Other observed neurological symptoms included depressed conscious state, motor aphasia and bulbar paresis, and progressive drowsiness. There was an association with cutaneous vasculitis, with CNS involvement often comorbid with this condition (72.7%, 8/11), including facial/lip/periorbital/limb edema, dilated nail bed capillaries, new ulcers on fingers/toes, and reticulated purpura. Among the nine cases reported previously, three were considered to have MAS/HLH complications (11–13); all three had hyperferritinemia and two had pancytopenia and fever. Most patients (77.7%, 7/9) had initial serum CK > 1,000 U/L. Only three underwent MSA analysis; one tested negative and two tested positive for anti-NXP2 antibody (i.e., in our center). Most cerebrospinal fluid examinations were negative (77.7%, 7/9); only two showed a slight increase in protein concentration. Non-invasive cerebral imaging (e.g., MRI) demonstrated a relatively high positivity rate (87.5%, 7/8), while MRA showed no positive findings in the cases examined. Brain biopsies were performed in four cases. Two showed pathological evidence of cerebral vasculitis (8, 10) and one indicated cerebral infarction (10). One patient with MAS complications exhibited brain tissue macrophage infiltration (11). Among the previous cases, despite receiving high-dose steroids or combined immunosuppressive therapy, most nevertheless had fatal outcomes, with a mortality rate of 66.6% (6/9). As described above, both patients at our center survived.

## 4 Discussion

In conclusion, CNS involvement in JDM represents a significant and under-recognized complication. Improved recognition, early diagnosis, and appropriate management of these neurological complications are essential for optimizing patient outcomes. Through our retrospective analysis and literature review, we aimed to describe the epidemiology, clinical manifestations, and management strategies for CNS involvement in JDM, ultimately guiding future research and clinical practice in this field.

In our review of patients diagnosed with JDM in our center, there were two with CNS involvement, indicating an incidence rate of 1.03% (2/193) in JDM. Although rare, it can be life-threatening. In the previously reported nine pediatric cases, despite treatment, up to 66.6% (6/9) of patients ultimately had a fatal outcome. Therefore, it is crucial to pay significant attention to, and provide proactive intervention in, such cases.

To date, the mechanism behind the CNS involvement in JDM remains unclear. Multiple pieces of evidence suggest that vascular involvement is a major pathological event in the organs affected by JDM (21–24). Among the cases we reviewed, four underwent brain biopsies; two showed direct pathological evidence of CNS vasculitis (CNSV) (8, 10) and one indicated cerebral infarction (10), further confirming the significant role of vascular lesions in JDM with CNS involvement. Additionally, one brain biopsy of a patient with JDM accompanied by MAS showed macrophage infiltration into brain tissue (11), suggesting that cerebral MAS may be another factor in the pathogenesis of CNS involvement in JDM.

In the two patients described from our center, seizures occurred in the active stage of JDM. Both patients presented with severe muscle weakness and active cutaneous vasculitis manifestations. We were careful to exclude diseases that might cause neurological manifestation, including CNS infections, hypoperfusion/hypertensive encephalopathy, electrolyte and metabolic abnormalities, hypercoagulability, and autoimmune encephalitis. Therefore, we considered CNS involvement related to JDM. Based on the results of previous brain biopsies, we speculate that CNS involvement in JDM may be caused by secondary CNSV or cerebral MAS. These lesions are likely a result of abnormal activation of the immune system and inflammatory responses. Immunosuppressive therapy can improve these lesions by suppressing inflammation and immune reactions, leading to favorable clinical responses. These patients’ favorable clinical responses to immunosuppressive therapy also indirectly validated our diagnoses.

However, our cases cannot confirm the occurrence of CNSV. The diagnostic criteria for CNSV include: recent history or presence of an acquired neurological deficit unexplained by other causes; evidence of vasculitis in a CNS biopsy specimen; or cerebral angiogram with changes characteristic of vasculitis (25, 26). Due to the invasiveness of brain biopsy, it was not performed in our patients. Additionally, MRA being normal in our patients does not rule out the possibility of JDM-related CNSV, as MRA has a low diagnostic detection rate in this condition. Hence, we cannot provide definitive confirmation but also cannot rule out the presence of CNSV in our cases.

HLH is a life-threatening systemic hyperinflammatory syndrome. HLH in the context of rheumatologic diseases is regarded as secondary HLH, commonly known as MAS. It is characterized by fever, elevated ferritin and other markers of systemic inflammation, inappropriately low blood cell counts, disseminated intravascular coagulopathy, hepatitis, CNS inflammation, and high risk for progression to multiple organ dysfunction, shock and often death. It typically arises as a complication of rheumatic diseases like systemic juvenile idiopathic arthritis and adult-onset Still’s disease (27). MAS is extremely rare in JDM (28), to the extent that a classification criteria for MAS in JDM has yet to be established. In our literature review, three patients were considered to have MAS/HLH complications. Among these, all had hyperferritinemia, and two presented with pancytopenia and fever. Since both of our patients presented with hyperferritinemia along with thrombocytopenia, we also suspected the possibility of JDM combined with MAS. But neither patient exhibited fever, and there was insufficient evidence to consider a diagnosis of MAS. Nevertheless, this hypothesis cannot be ruled out, and is supported by the prompt response to calcineurin inhibitor (tacrolimus) in Case 2. Thus, we believe that there is indeed a high possibility of MAS contributing to the neurological involvement.

Our review of studies reporting comorbid JDM and CNS involvement over the past 30 years identified nine patients. Adding the two from our center, the median age for disease onset was 10.5 years (range 4–17 years), and the male-to-female ratio was 6:5. Seizures were the most common clinical manifestation of CNS involvement and often occurred together with cutaneous vasculitis, especially within 10 months after diagnosis. This suggests that active cutaneous vasculitic lesions may be a high-risk factor for concurrent CNS vasculopathy, possibly attributed to vascular dysfunction. The previous literature suggests that the presence of cutaneous vasculitic lesions can also be a predictive sign for diseases associated with severe systemic manifestations (29). Thus, seizures and active cutaneous vasculitic lesions in patients with JDM should raise clinical suspicion of CNS involvement.

Herein, most patients had initial serum CK levels >1,000 U/L. Consistent with this, a retrospective analysis by Van Rossum et al. found that patients in the poor prognosis JDM/polymyositis group had high serum CK (30). It is therefore recommended that caution be used when high initial CK is detected, as this may indicate a more severe condition.

MSA, a specific serum antibody for JDM, is closely associated with specific organ involvement or specific clinical subtype presentations (6). While research on MSA specifically related to CNS involvement remains extremely limited, among the nine previous case reports, only one underwent MSA testing (and the result was negative) because MSA testing was not widely available before 2017 (31). In our center, both patients were anti-NXP2 antibody-positive. Previous studies on anti-NXP2 antibody-positive JDM have primarily focused on clinical features like calcinosis, severe muscle involvement, gastrointestinal vasculitis, and swallowing difficulties, with poor prognosis (22, 32–34). The CNS involvement observed in our two patients who were anti-NXP2 antibody-positive represents a relatively new phenotype. In our center, there seems to be a relatively higher concentration of CNS involvement in patients with anti-NXP2 antibody-positive JDM, with a frequency of 4.54% (2/44) among the evaluated cases. However, this sample is limited and more data are needed to determine whether there is a significant association between CNS involvement and MSA. MSA has also emerged as a valuable biomarker for patients with equivocal clinical characteristics of JDM. Case 1 presented with rare skin manifestations, including asymmetric upper limb edema and reticular purpura, without typical dermatomyositis rash. We further confirmed our diagnosis through MSA testing. Therefore, we also recommend MSA testing to assist in diagnosis of patients with equivocal clinical JDM characteristics, assess clinical phenotypes, and predict prognosis.

These cases demonstrated a relatively high positive detection rate on MRI, about 87.5% (7/8). These positive results may include changes such as local ischemia or infarction, microbleeds, cerebral edema, and vascular lesions. Considering the important roles of various MRI sequences (e.g., T2, FLAIR for assessing edema and ischemic changes, T1 for evaluating parenchyma and meninges, DWI and ADC for early identification of cerebrovascular accidents and differentiating cerebral edema types (35) we recommend performing a comprehensive MRI examination including each of these sequences to assist in the differential diagnosis.

As mentioned above, cerebral angiogram plays a crucial role in the diagnosis of CNSV. That MRA showed no positive findings in the patients examined may be related to the fact that most MRA examinations did not include vessel wall imaging (VWI). When VWI is included, the diagnostic accuracy of MRA in identifying vasculitis increases from only 8.3% to 95.8% (36). Thus, we assert that cerebral MRA (preferably including VWI) has a significant role in identifying cerebral vasculitis. Invasive examinations like CSF collection and brain biopsy can provide valuable information for diagnosis. In these patients, CSF examinations were mostly negative, with a few showing slightly elevated protein. While brain biopsy can play a significant role in confirming the diagnosis, it is largely limited in clinical applications because of its invasiveness.

When JDM involves important organs, it is referred to as severe JDM (37). It is currently recommended that IVMP pulse therapy be used to treat severe active JDM, and that intravenous injections of IVIG and/or IVCY treatment be combined as early as possible to control this condition (38). Both of our patients were treated accordingly, but were still progressing in of their conditions. A few reports have shown that PE can help improve systemic symptoms and laboratory indicators in critically ill children with JDM (39–41). In their 2011 review and analysis of 33 patients with systemic autoimmune diseases (SAIDs) who were receiving PE therapy, Pons-Estel et al. concluded that this is an effective treatment option in severe SAIDs manifestations (42). Both of our patients also underwent PE therapy, after which Case 1 showed symptom relief and improvement in indicators, while Case 2 showed no sign or symptom improvements. To salvage the treatment for Case 2, two courses of IVMP pulse therapy were administered, along with oral tacrolimus for potential MAS. It is noteworthy that both patients received necessary life support measures (e.g., prolonged mechanical ventilation and, for Case 2, tracheostomy). Consequently, both patients survived. In the previously reported cohort of nine pediatric patients, the mortality rate was 66.6% (6/9). Our patients’ survivals might be attributed to our timely, proactive approaches, including interventions like PE, IVMP pulse therapy, and life support therapy, including treatments for potential MAS. This may be a novel effective treatment for severe active JDM and refractory cases. It is also worth noting that both patients experienced secondary infections, highlighting the need for vigilant monitoring, including for opportunistic infections, when intensifying immunosuppressive therapies.

## 5 Conclusion

To our knowledge, this is the first case report of CNS involvement in anti-NXP2 antibody-positive JDM. JDM with CNS involvement, a rare but potentially life-threatening complication, should not be overlooked, especially during the 10 months after diagnosis. It usually manifests as seizures, often accompanied by active cutaneous vasculitis. In patients with the presence of hyperferritinemia and thrombocytopenia should raise caution for potential MAS. Calcineurin inhibitors can be considered as one of the treatment options and may provide certain benefits. In refractory cases, timely implementation of salvage therapies like PE and IVMP pulse therapy, and necessary life support therapies, may significantly impact patient outcomes. MSA has emerged as a valuable laboratory indicator for patients with equivocal clinical JDM characteristics. However, further data are needed to confirm the association between CNS involvement in JDM and MSAs.

## Data Availability

The original contributions presented in the study are included in the article/Supplementary Material, further inquiries can be directed to the corresponding author.
